# Emerging Strategies and Integrated Systems Microbiology Technologies for Biodiscovery of Marine Bioactive Compounds

**DOI:** 10.3390/md12063516

**Published:** 2014-06-10

**Authors:** Javier Rocha-Martin, Catriona Harrington, Alan D.W. Dobson, Fergal O’Gara

**Affiliations:** 1 BIOMERIT Research Centre, School of Microbiology, University College Cork, National University of Ireland, Cork, Ireland; E-Mails: jrocha@ucc.ie (J.R.-M.); catriona.harrington@ucc.ie (C.H.); 2 School of Microbiology, University College Cork, National University of Ireland, Cork, Ireland; E-Mail: a.dobson@ucc.ie; 3 Marine Biotechnology Centre, Environmental Research Institute, University College Cork, National University of Ireland, Cork, Ireland; 4 School of Biomedical Sciences, Curtin University, Perth, WA 6102, Australia

**Keywords:** marine bioactive compounds, metagenomics, synthetic biology, biocatalyst discovery, metaproteomic, dereplication, omic approaches

## Abstract

Marine microorganisms continue to be a source of structurally and biologically novel compounds with potential use in the biotechnology industry. The unique physiochemical properties of the marine environment (such as pH, pressure, temperature, osmolarity) and uncommon functional groups (such as isonitrile, dichloroimine, isocyanate, and halogenated functional groups) are frequently found in marine metabolites. These facts have resulted in the production of bioactive substances with different properties than those found in terrestrial habitats. In fact, the marine environment contains a relatively untapped reservoir of bioactivity. Recent advances in genomics, metagenomics, proteomics, combinatorial biosynthesis, synthetic biology, screening methods, expression systems, bioinformatics, and the ever increasing availability of sequenced genomes provides us with more opportunities than ever in the discovery of novel bioactive compounds and biocatalysts. The combination of these advanced techniques with traditional techniques, together with the use of dereplication strategies to eliminate known compounds, provides a powerful tool in the discovery of novel marine bioactive compounds. This review outlines and discusses the emerging strategies for the biodiscovery of these bioactive compounds.

## 1. Introduction

The marine habitat continues to be a source of unique natural products used for pharmaceutical and biotechnological applications [1]. In fact, the Roman philosopher Plinius first described the use of marine natural organisms such as sponges in medicinal applications, such as for the treatment of wounds, sunstroke and infections, around 2000 years ago [2]. More than 20,000 structurally diverse marine natural products have been isolated from ocean life forms such as sponges, ascidians, aplysia, algae, corals, bryozoan, worms, sea-hares, sea-cucumbers, fish species and microorganisms (Table 1 and Table 2) [3,4]. Marine microorganisms continue to be a major focus of many natural product research efforts, with a 10% increase in the number of compounds reported from 2011 to 2012 [4]. More than 70% of the Earth’s surface is covered by the ocean which contains a vast collection of diverse microbial communities, all interacting with each other and with the environment around them. In fact, it is estimated that the ocean contains the highest percentage of prokaryotic cells on Earth, with a reported 3.67 × 10^3^^0^ cells [5]. It is believed that specific physiochemical properties of the marine environment, such as pressure, temperature, pH, osmolarity, and uncommon functional groups (such as isonitrile, dichloroimine, isocyanate, and halogenated functional groups) may result in the production of bioactive substances with different properties from those found in terrestrial habitats (Table 1 and Table 2) [3,6,7]. 

**Table 1:** Examples of marine bioactive compounds or derivatives approved by U.S. Food and Drug Administration (FDA) or in clinical trial with a bacterium predicted biosynthetic cluster. Sources: [3,8,9].

Compounds	Natural Product or Derivative	Collected Source Organism	Activity	Clinical Status
Salinosporamide A (Marizomib)	Natural product	Bacterium	Antitumor	Phase I
Plitidepsin (Aplidin)	Natural product	Tunicate	Antitumor	Phase III
Bryostatin 1	Natural product	Bacterium	Antitumor/Anti-Alzheimer	Phase II
Cytarabine	Derivative	Sponge	Antitumor	Approved
Vidarabine	Derivative	Sponge	Antiviral	Approved
Eribulin Mesylate	Derivative	Sponge	Antitumor	Approved
Trabectidin (ET-743)	Natural product	Tunicate	Antitumor	EU approved

The marine environment can also be considered relatively unexplored in relation to the presence of enzymatic activities which can be found. Moreover, a marine enzyme may carry more novel chemical and stereochemical properties than those found in terrestrial environments [7]. Marine microorganisms are considered to be the primary source of marine enzymes. In particular, there is a huge interest in extremophiles, as the robustness of their biocatalysts have allowed them to adapt in order to survive and indeed thrive in extreme ecological niches such as in high or low temperatures, extremes of pH, high salt concentrations and high pressure. These characteristics, along with substrate specificity, are evolved properties that are linked to the metabolic functions of the enzymes and to the ecological asset related to natural sources [7]. This biodiversity has greatly increased interest in this field.

Sponges are the dominant source of novel natural products in the marine environment. In fact, more novel compounds are identified per year from marine sponges than from any other invertebrate phylum [10]. In a recent review by Hu *et al.* [11], approx. 3500 (out of 12,000) novel marine natural products were identified from marine sponges (such as novel anti-inflammatory agents, anticancer agents and antibiotics) between 1985 and 2008, which further emphasizes their importance in drug discovery and bio-prospecting [11]. Sponges are believed to be highly complex selective feeders that house a dense and diverse arsenal of symbiotic bacteria (bacterial phyla such as Actinobacteria, Acidobacteria, Chloroflexi, Planctomycetes, Proteobacteria, Nitrospira, Cyanobacteria, Bacteriodetes, Verrucomicrobia and Poribacteria), archea and unicellular Eukaryotes within their mesohyl tissue [12]. Fungi and microalgae are also known to inhabit and form symbiotic relationships with marine sponges [13]. These symbiotic microorganisms can represent approximately 35%–40% of the total sponge volume, densities which are much higher than the surrounding sea water [14]. As sessile organisms, sponges rely on a barrage of chemical entities, generally produced by their associated microorganisms, to defend against disease and to gain a competitive advantage within the marine ecosystem [12].

**Table 2:** Examples of recently identified biocatalysts from marine environment.

Enzyme	Source	Screening Technologies	Reference
Laccase	Metagenome	Sequence-based	[15]
Esterase	Metagenome	Function-based	[16]
Fumarase	Metagenome	Sequence-based	[17]
α-amilase	*Bacillus subtilis* S8–18	Function-based	[18]
Glycoside hydrolase	Metagenome	Function-based	[19]
Baeyer-Villiger monooxigenase	*Stenotrophomonas maltophila* PML168	Function-based	[20]
Cellulase	*Marinimicrobium* sp.	Function-based	[21]
Dehalogenase	*Pseudomonas stutzeri* DEH130	Function-based	[22]
Aldehyde reductase	*Oceanospirillum* sp.	Genome-based	[23]
Lipase	*Bacillus smithii* BTMS11	Function-based	[24]
Dehalogenase	*Psycromonas ingrahamii*	Genome-based	[25]
Xylanase	*Streptomyces viridochromogenes*	Function-based	[26]

In this review, strategies for the discovery of novel marine bioactive compounds are discussed, in particular, how modern molecular biology approaches (“omic” approaches) can be integrated with microbiology techniques to provide new and better options for the mining of novel bioactive compounds and enzymes from marine bacteria.

## 2. Culture Dependent and Independent Isolation of Marine Microorganisms

### 2.1. Culture Dependent Analysis of Structure and Function of Marine Microbial Communities

Isolation and cultivation of a novel marine microorganism presents a bottleneck in the discovery of new marine natural products. In many natural environments, including the marine environment, bacterial numbers estimated by direct counts using microscopic techniques are generally several orders of magnitude higher than estimations by Colony-Forming Units (CFUs) using standard culture techniques. Only between 0.001% and 2% of bacterial cells can form colonies by conventional plate cultivation due to the large number of “Viable But Not Culturable” (VBNC) strains [27]. This is therefore the major limitation of culture dependent techniques [28].

In recent years, however, huge improvements have been made in the production of both culture media and cultivation procedures. These have been devised to mimic natural environments in terms of nutrients (composition and concentration), pressure, pH, oxygen gradient, *etc.* to maximize the cultivable fraction of microbial communities [29,30,31,32,33,34]. With these improvements, some previously VBNC species can now be grown by the refinement of classical approaches or by the use of advanced techniques (e.g., High Throughput Screening (HTS), diffusion chamber system, encapsulation method, soil substrate membrane system, filtration method, density-gradient centrifugation, extinction dilution and Fluorescence-Activated Cell Sorting (FACS)), which are discussed in detail in several published reviews, focused on this topic [35,36,37,38].

### 2.2. Culture Independent Analysis of Structure and Function of Marine Microbial Communities

**Figure 1:**
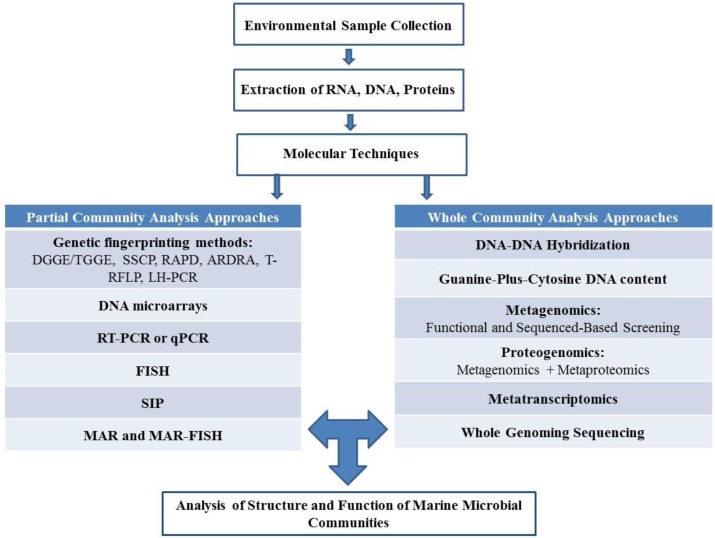
Culture independent analysis of structure and function of marine microbial communities.

In the past, the identification of the number of bacteria within a sample was completed using standard microscopy. However, recent advances in DNA/RNA-based techniques and sequence technologies have allowed for the identification and characterization of the diversity and function of bacteria within a microbial community without the use of laborious microscopy techniques [39]. Thanks to these advances, over the last few decades, tremendous progress has been made in the field of microbial ecology. These techniques have been classified into two major categories: partial community analysis approaches and whole community analysis approaches (Figure 1) [39]. 

#### 2.2.1. Partial Community Analysis Approaches

Partial community analysis approaches generally involve polymerase chain reaction (PCR)-based methods where total DNA/RNA extracted from an environmental sample is used in the characterization of microorganisms. Clone library construction is the most widely used method to analyze PCR products amplified from an environmental sample [40,41,42,43]. Using this technique, the bacterial communities in three sponges—namely *Hymeniacidon perlevis*, *Ophlitaspongia papilla* and *Polymastia penicillus*—from the Atlantic coast of Portugal were characterized, confirming a unique and diverse microbial community from 10 different bacterial phyla [42]. Likewise, this method was used in the seasonal characterization of the bacterial community of the Irish coastal water sponge *Hymeniacidon perlevis*, with three bacterial phyla detected in summer, and four detected in spring [44].

Genetic fingerprinting—another technique commonly used in partial community analysis—generates a profile of a microbial community based on the direct analysis of PCR products amplified from environmental DNA, generally using 16S rRNA gene analysis [45]. These techniques include DGGE (denaturing-gradient gel electrophoresis)/TTGE (temperature-gradient gel electrophoresis) [46], SSCP (Single-Strand Conformation Polymorphism), RAPD (Random Amplified Polymorphic DNA) [47,48], ARDRA (Amplified Ribosomal DNA Restriction Analysis) [49], T-RFLP (Terminal Restriction Fragment Length Polymorphism) [50], LH-PCR (Length Heterogeneity PCR) and RISA (Ribosomal Intergenic Spacer Analysis) (Figure 1) [51,52].

To increase the throughput of the detection of microorganisms within complex samples, DNA microarrays can be used in parallel for the detection of multiple species. DNA microarrays used in microbial ecology can be classified into two groups depending on the probes used: (1) 16S rRNA gene microarrays [40,53,54]; and (2) functional gene arrays (FGA) to detect specific metabolic groups of bacteria [54,55].

RT-PCR (Real Time-PCR) (also known as qPCR (Quantitative-PCR)) has also been applied to quantify functional genes within the environment [56,57].

Fluorescence *in situ* hybridization (FISH) has enabled the identification of bacterial taxa and their spatial distribution [58,59,60]. In FISH analysis, probes are designed to target a particular 16S rRNA gene within a sample. Sequence analysis of 16S rRNA genes is commonly used in microbial ecological studies. However, despite its highly conserved nature, the 16S rRNA gene does not provide sufficient resolution at species and strain level [61]. Another drawback of FISH analysis is that, although bacterial cell numbers can be estimated using this approach, non-viable cells are still included and hence results can be misleading [62].

RNA extracted from environmental samples provides more valuable information than DNA in revealing active microbial communities *versus* dormant microbial communities [63]. This is due to the fact that rRNA and mRNA are considered to be indicators of functionally active microbial populations. The amount of rRNA in a cell is approximately correlated with the growth activity of bacteria, and the mRNA of functional genes allows the detection and identification of bacteria expressing key enzymatic activities under determined conditions [64]. The comparative and quantitative analysis of expressed rRNA and key enzymatic genes can be useful in obtaining information about bacterial groups responsible for processes such as methane oxidation, denitrification and nitrification [63].

More advanced partial community analysis approaches include stable isotope probing (SIP) [65,66], where active microbial communities that utilize the labeled biomolecules (e.g., DNA, RNA, phospholipid fatty acids) during growth incorporate isotopes within their biomass. Subsequently, the labeled biomolecules are separated from their biomass by different biochemical methods, and the phylogenetic identity of microorganisms metabolizing the substrate can be established using molecular techniques. SIP can be combined with microarray technology for monitoring the substrate uptake profiles and establishing the taxonomic identities of active microbial communities [67]. Other techniques, such as microautoradiography (MAR) rely on the fact that metabolically active cells utilizing radiolabeled substrate can be visualized by exposure to radiation-sensitive silver halide emulsion [60]. The combination of MAR with FISH (MAR–FISH) allows the simultaneous phylogenetic identification of active cells that consume the radioactive substrate [68]. 

#### 2.2.2. Whole Community Analysis Approaches

Whole community analysis approaches offer a more comprehensive view of the genetic diversity within a community compared to PCR-based molecular approaches, which target only a single or a few genes [39]. These approaches attempt to analyze all the genetic information present in total DNA extracted from an environmental sample or pure culture. The techniques in question include DNA-DNA hybridization kinetics, guanine-plus-cytosine (G + C) DNA content, metagenomics and whole-microbial genome sequencing as well as emerging “omics” technologies which will be discussed later.

In DNA–DNA hybridization, the total DNA extracted from an environmental sample (eDNA) is denatured and subsequently incubated under conditions that allow hybridization to occur. The rate of DNA hybridization is correlated with the genomic diversity present in the sample [69].

Another technique known as guanine-plus-cytosine (G + C) is based on the separation of bacterial groups based on the varying G + C content of their DNA [70]. Total community DNA is physically separated (by density-gradient centrifugation) into highly purified fractions, each representing a specific percentage G + C content that can be analyzed by additional molecular techniques such as DGGE/ARDRA to better assess total community diversity [71].

Through metagenomic tools, we can analyze the microbial diversity of an environmental sample, bypassing the need for a culture based approach and thus allowing the study of VBNC in the laboratory [72]. Metagenomic library construction involves the following steps: isolation of eDNA, shotgun cloning of random DNA fragments into a suitable vector, transformation of the clones into a host bacterium and screening for positive clones. Metagenomic libraries can be screened either by sequence-driven metagenomic analysis that involves massive high-throughput sequencing, or by functional screening of expressed phenotypes. High throughput screening (HTS) of large libraries enables the identification of individual clones containing regions of DNA encoding potentially novel genetic clusters. PCR amplification of the DNA segment within a specific clone then allows for the identification of the gene responsible for the observed phenotype. In depth analyses of the bacterial populations associated with marine sponges have been completed using metagenomic analysis. These studies have identified a large amount of species diversity within marine samples [73,74]. More recently, the emergence and development of the Next Generation Sequencing (NGS) platforms (454 from Roche, SoLiD from ABI or Solexa from Illumina) has helped to improve the quality and speed of the processor DNA [75]. This technology, for example, has facilitated the generation of an exponential number of sequencing reads from metagenomic DNA samples and has enabled the identification of members of the “rare-biosphere” [76]. As the major drawback to the metagenomic approach is the lack of reference genomes and sequences, the vast increase in data and tools now becoming available should gradually minimize this problem [77]. Another problem frequently encountered in metagenomic studies is the availability of suitable hosts for the heterologous expression of these unknown DNA sequences in order to ascertain the maximum information from functional metagenomic analyses [77]. Once the sequences are obtained, they are aligned and assembled into finished sequences using specialized computer programs such as MEGAN (MEtaGenome ANalyzer) [78]. These sequences can be annotated into open reading frames (ORFs) to predict the encoded proteins (functions), using web-based programs such as Rapid Annotation using Subsystem Technology (RAST) [79,80]. The assignment of the taxonomic status to these sequences requires the use of softwares such as Metagenomic And rDNA Taxonomy Assigner (MARTA) [81] which is available at the MARTA web site [82], or Genomic Signature based Taxonomic Classifier (GSTaxClassifier) [83] which is available at GSTaxClassifier web site [84]. The exploration of microbial communities through whole-microbial genomes is an exhaustive and integrated approach to understand microbial ecology and function. The enormous amount of data gathered from genome sequencing programs is deposited in searchable databases that could be mined using various powerful bioinformatic tools available, such as Integrated Microbial Genomes with Microbiome Samples (IMG/M) web server [85,86] for evolutionary studies, comparative genomics, and proteomics, CAMERA (Community CyberInfrastructure for Advanced Marine Microbial Ecology Research and Analysis) [87,88], MG-RAST for phylogenetic and functional analysis of metagenomes [89,90]. For example, Microbial Genomes Resources at the National Center for Biotechnology Information (NCBI) [91] is a public database for prokaryotic genome sequencing projects. The Genome Online Database (GOLD) [92] is another database resource for comprehensive information regarding complete and ongoing genome projects, metagenomes and metadata. As of 22 February 2014, the GOLD database held 3012 completed and published genomes, 2690 bacterial and 171 archaeal genomes, and 17,564 bacterial genomes in progress [93].

#### 2.2.3. Emerging “Omics” Technologies to Analyze the Structure and Function of Marine Microbial Communities

The emerging “omics” technologies, such as metaproteomics, proteogenomics and metatranscriptomics, have potential applications in microbial ecology in the identification of novel functional genes, new catalytic enzymes or metabolic pathways, and in the use of biomarkers to monitor the changes in a microbial community over time. Therefore, these technologies allow us to link the structure and function in microbial communities.

Metaproteomics allow us to determine which proteins were synthesized by microorganisms at the time of sample collection. This allows the reconstruction of the most important metabolic pathways and microbial processes in the configuration of an ecosystem [94]. In metaproteomics, the correct bioinformatic assignment of spectrometrically determined peptide masses of environmental origin is highly dependent on the available genomes of closely related species in the database [95]. This approach has been performed on the microbial population of the world’s oceans [96,97,98]. For example, Cavicchioli *et al.* [98] have examined the proteome of microbial communities in coastal Antarctic waters for differences in functional processes (transport, metabolism and energy generation processes) occurring between summer and winter. Recently, Steen *et al.* [99] used a proteomic approach to study anaerobic methanotrophic archaea and sulfate-reducing bacteria in cold marine sediments, investigating expressed functions of the community in combination with assembled sequences from the metagenome. 

In metaproteomics, protein sequences can be identified with confidence only if they have significant homology to existing proteins in available databases. However, in most environmental proteomic surveys, proteins are only distantly related to known database sequences. These limitations have been overcome by combining metaproteomic and metagenomic approaches together under the name of proteogenomics [100]. Christie-Oleza *et al.* [101] used whole-cell, Matrix-Assisted Laser Desorption/Ionization Time-Of-Flight Mass Spectrometry (MALDI-TOF MS) for the screening of natural marine isolates obtained from the coastal waters of the Western Mediterranean Sea. In order to make this technique accessible for environmental studies, they proposed to (1) define biomarkers that will always show up with an intense m/z (mass number/charge number ratio) signal in the MALDI-TOF spectra (HU, a DNA binding protein, and the ribosomal proteins, L29 and L30, were proposed as the most robust biomarkers within the Roseobacter clade) and (2) create a database with all the possible *m/z* values that these biomarkers can generate to screen new isolates. The molecular weights of the three proposed biomarkers, as for other conserved homologous proteins, vary due to sequence variation above the genus level. Therefore, the expected m/z values were calculated for each one of the known *Roseobacter* genera and tested *versus* standard sequencing methods (such as 16S rDNA sequencing). Another interesting approach, although the samples are not from marine source, is described by Singer *et al.* [102]. Here, the authors carried out metagenomic and proteogenomic analyses of a compost-derived bacterial consortium adapted to switchgrass at elevated temperatures with high levels of glycoside hydrolase activities. Singer *et al.* identified genes for lignocellulose processing and metabolic reconstructions, and suggested *Rhodothermus*, *Paenibacillus* and *Gemmatimonadetes* as key groups for degrading biomass. *Thermus* was identified as a group that may especially metabolize low molecular weight compounds. Partial genomes were also reconstructed for a number of lower abundance thermophilic *Chloroflexi* populations. These studies indicate that there are unexplored proteins (potential source of thermophilic enzymes) with important roles in bacterial lignocellulose deconstruction. A similar approach can be used in the marine environment to discover interesting biocatalysts.

Metatranscriptomics enables us to identify activities and investigate gene regulation in complex microbial communities, both for experimental studies of genetically manipulated systems and for descriptive studies of gene expression, without the necessity of presupposing which genes should be targeted [103,104,105]. Thus, metatranscriptomics is particularly suitable for measuring changes in gene expression and regulation with respect to changing environmental conditions. Oruga *et al.* [103] constructed two libraries from samples of marine microorganisms taken from Hiroshima bay in Japan. Functional analysis of genes revealed that a small number of gene groups, namely ribosomal RNA genes and chloroplast genes, were dominant in both libraries. Taxonomic distribution analysis of the libraries suggests that Stramenopiles form a major taxon that includes diatoms. The combination of metagenomic and metatranscriptomic approaches has proven to be an effective strategy in deciphering the phylogenetic composition, and metabolic approaches have proven efficacious in deciphering the phylogenetic composition, and the metabolic pathways of marine microbial communities [105,106,107]. Here, the metagenomic approach provides information on the metabolic potential of a microbial community and the taxonomic composition, while the metatranscriptomic approach provides information about the actual metabolic activities of the community at a concrete place and time, and how those activities change in response to biotic interactions or environmental forces. For example, in a recent study, using coupled metagenomic and metatranscriptomic analysis of the microbial communities in the deep-sea water of the North Pacific Ocean [107], it was determined that, within the prokaryotic community, bacteria are dominant over archaea in both metagenomic and metatranscriptomic data pools. On one hand, the main compositional change in prokaryotic communities in the deep-sea water, compared with the reference Global Ocean Sampling Expedition (GOS) surface water, was the emergence of the archaeal phyla Crenarchaeota, Euryarchaeota, Thaumarchaeota, bacterial phyla Actinobacteria, Firmicutes, and bacterial sub-phyla Betaproteobacteria, Deltaproteobacteria, and Gammaproteobacteria. On the other hand, decreased levels of the bacterial phyla Alphaproteobacteria and Bacteroidetes were observed. Photosynthetic Cyanobacteria were present in all four metagenomic libraries and two metatranscriptomic libraries studied. Functional analysis indicated that the groups with adaptive advantages against high pressure, low nutrient concentration and multidrug resistance to antimicrobials increase their presence in the deep-sea water. All these adaptations are indicative of a defensive lifestyle [107]. 

### 2.3. Culture Dependent and Independent Analysis. Combined or Separately?

The limitations of both strategies are well known (involving the isolation and cultivation of a novel microorganism in the case of culture methods, and the DNA extraction and PCR efficiency in the case of independent analysis), however, numerous studies in the literature have overcome this by combining the use of both culture dependent and independent approaches in order to strengthen their results [108,109,110,111,112]. For example, the microbiota of the marine demosponge *Crambe crambe* was examined using these combined cultivation-dependent and cultivation-independent molecular approaches [109]. Authors found a low microbial diversity, which is dominated by a thus far uncharacterized bacterium belonging to a betaproteobacterial clade that is specific to sponges and corals. The most frequently isolated genus of 107 isolated from three *C. crambe* individuals was *Pseudovibrio*, followed by *Microbulbifer*, *Bacillus* and *Ruegeri*. Phylogenetic analysis based on 16S rRNA gene sequence was then used to determine the relative abundance of these isolates to avoid cultivation bias. These analyses showed that *Pseudovibrio* spp., *Microbulbifer* spp., *Ruegeria* spp. isolated were closely related to microorganisms previously isolated from marine sponges and other marine invertebrates by cultivation dependent and independent methods. Each 16S rRNA gene sequence obtained from the isolates was compared to pyrosequencing libraries generated. This comparison revealed that only a low number of the isolates (around 0.4% of the total microbiota) were also detected in the 16S rRNA gene pyrosequencing libraries, while others were absent. One exception was *Microbulbifer* spp. isolates, which were present in a high number of pyrosequencing reads only in one *C. crambe* individual and absent in the other individual. The fact that they were found in relatively high numbers in the individual from which it was isolated shows a certain degree of overlap between cultivation dependent and independent approaches. *Pseudovibrio*, which was the most frequently isolated genus, was detected in only very low numbers in both 16S rRNA gene sequence datasets. Öztürk *et al.* suggested that the potential production of antibacterial compounds by *Pseudovibrio* spp. might be one of the reasons why they overgrow in enrichment cultures. 

On the other hand, a study of hydrocarbonoclastic bacteria in various oily habitats in Kuwait by Al-Awadhi *et al.* [108] showed that bacterial identities varied dramatically depending on the analytical approach used. In this work, the results obtained by molecular analysis were compared with the results obtained by culture-dependent analysis of a previous study [113]. In contrast to the culture-dependent technique, primers used in the molecular analysis preferentially amplified the 16S rDNA of hydrocarbonoclastic bacteria in the total environmental genomic DNA of all the studied samples. On the other hand, molecular analysis failed to reveal members of the phylum *Actinobacteria*, which conversely were identified via the culture-based approach. In view of these results, the authors recommended that the two analytical approaches should be used simultaneously because their combined results would reflect the bacterial community composition more precisely than either of them could do alone.

Moreover, even genomic analysis has been used to guide cultivation efforts. For example, in a study performed by Tripp *et al.*, the genomic analysis of the SAR11 α-proteobacterial clade showed that the clade was deficient in assimilatory sulphate reduction genes [114]. Therefore, the complementary use of cultivation-dependent and cultivation-independent approaches would provide a more robust screening strategy. Molecular techniques that allow us to bypass the need for isolation and cultivation are highly desirable for in depth characterization of environmental microbial communities. However, culture dependent techniques provide more information about the physiological and metabolic characteristics of bacteria and their communities, and their responses to environmental changes.

## 3. Strategies for Marine Natural Compounds Discovery from Marine Microbial Communities

Different screenings approaches have been exploited in the identification of novel natural compounds including conventional screening, genomics, metagenomics, combinatorial biosynthesis and synthetic biology.

### 3.1. Conventional Screening Methods: Bioactivity-Guided Screening

Bioactivity screening is based on the direct detection of the activity (e.g., antibacterial, antifungal, antitumor, antiviral activity) using the culture supernatant or extract of cell pellet [115,116,117,118,119]. In one particular study, Tong *et al.* [115] collected 38 microbial extracts from Hawaiian coastal waters, which were then evaluated for their antiviral activity against four mammalian viruses, namely herpes simplex virus type one (HSV-1), vesicular stomatitis virus (VSV), vaccinia virus and poliovirus type one (poliovirus-1) using an *in vitro* cell culture assay. Nine of these microbial extracts showed antiviral activity, and three of the nine showed significant activity against the enveloped viruses. In this manner, the secosteroid 5α(*H*),17α(*H*),(20*R*)-beta-acetoxyergost-8(14)-ene was identified as a novel bioactive compound in these extracts. 

Dalisay and coworkers [120] investigated marine sediments collected in the temperate cold waters of British Columbia, Canada, as a valuable source of novel groups of marine-derived *Streptomyces* with antimicrobial activities. They performed culture dependent isolation from 49 marine sediments samples and obtained 186 *Streptomyces* isolates. Twenty-five percent of these isolates exhibited antimicrobial activity. Phylogenetic analyses of these isolates revealed four different clusters, with one in particular representing a novel species. Chemical analyses revealed structurally diverse secondary metabolites produced by marine-derived *Streptomyces*, including four new antibacterial novobiocin analogues. They conducted structure-activity relationship studies of these novobiocin analogues against methicillin-resistant *Staphylococcus aureus* (MRSA). This study revealed the importance of carbamoyl and OMe moieties at positions 3′ and 4′ of novobiose as well as the hydrogen substituent at position 5 of hydroxybenzoate ring for the anti-MRSA activity. In another interesting approach, Sanchez *et al.* [119] examined six species of fish through a combination of dissection and culture-dependent evaluation of intestinal microbial communities. Using a specific enrichment medium designed to isolate marine *Actinobacteria*, Sanchez and coworkers found three main clades that showed taxonomic divergence from known strains. Furthermore, several of these strains have been previously described as nonculturable. Microbial extracts from these strains exhibited a wide range of activities against Gram-negative, Gram-positive human pathogens, and several fish pathogens. Exploration of these extracts has identified the novel bioactive lipid sebastenoic acid as an anti-microbial agent, with activity against *S. aureus*, *Enterococcus faecium*, *Bacillus subtilis*, and *Vibrio mimicus*.

The repeated discovery of known compounds is a major limitation of existing assay methods, hence it is necessary not only to increase the availability of novel techniques to maximize the discovery of new compounds, but also to utilize dereplication strategies to avoid the discovery of known compounds. These strategies include the generation of antibiotic resistance markers and chemical profile analysis based on LC-NMR, LC-MS and HPLC-UV [121].

### 3.2. Genome-Guided Bioprospecting

Genomic sequencing of microorganisms in recent years has unveiled unprecedented insights into the biosynthetic potential of microorganisms, and thus the discovery of bioactive compounds has entered into the new postgenomic era. In recent years, the sequence data of microorganisms has been compiled into online databases such as Genomes On Line Database [92]. The development of the Microbial Genome Sequencing Project resulted in the sequence, assembly and annotation of 182 marine bacterial genomes, which are available at [122]. Due to these advances in bioinformatics, it is now possible to rapidly identify the gene clusters of bioactive compounds and predict their chemical structure *in silico* based on genomic information. For example, the genome sequencing of *Streptomyces* *griseus* IFO 13350 revealed 34 biosynthetic gene clusters involved in the biosynthesis of unknown or known secondary metabolites [123]. 

**Figure 2:**
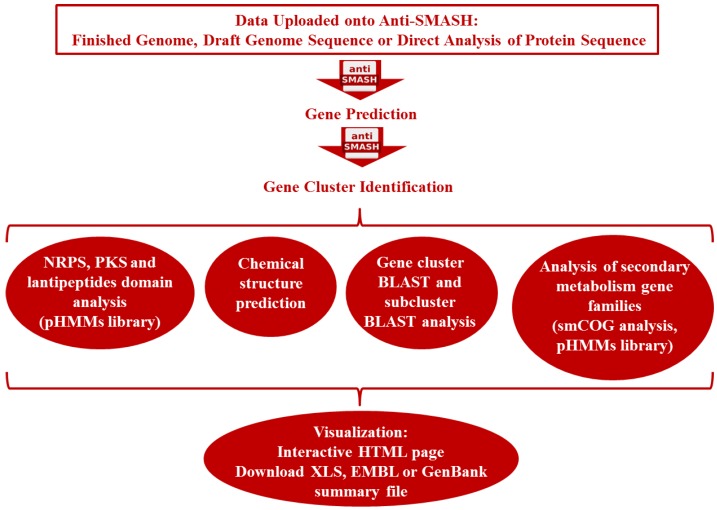
How AntiSMASH works. AntiSMASH is used for the detection of secondary metabolites. Abbreviations: pHMMs library: profile hidden Markov models composed of protein sequences of experimentally characterized biosynthetic enzymes; smCOGs: secondary metabolite-specific clusters of orthologous groups.

Along with advances in bioinformatics, genome sequencing has made it possible to rapidly identify the gene cluster of bioactive compounds and *in silico* predict their chemical structure based on genomics information. Development of software such as Antibiotics & Secondary Metabolite Analysis SHell (antiSMASH) (Figure 2), which allows the user to efficiently detect secondary metabolite gene clusters in the genomes of bacteria and fungi, has been a significant help to researchers [124,125]. AntiSMASH allows for the detection of biosynthetic gene clusters of secondary metabolites such as type I, II and III PolyKetides (PKs), Non-Ribosomal Peptides (NRPs), phosphoglycolipids, oligosaccharide antibiotics, phenazines, thiopeptides, homoserine lactones, phosphanates, furans, terpenes, ectoines, bacteriocins, lantibiotics, nucleosides, aminoglucosydes, aminocumarins amongst others. AntiSMASH also has the ability to partially predict types of compounds that can be produced if the gene cluster is completely functional [125]. 

Other useful bioinformatics tools for analyzing sequence data include: Bacteriocin Genome Mining Tool 3 (BAGEL3) for the detection and annotation of bacteriocin and ribosomally synthesized and posttranslationally modified peptides gene clusters [126,127]; Natural Product Domain Seeker (NaPDoS) for phylogenetic analysis of PoliKetyde Synthase (PKS) ketoreductases and Non-Ribosomal Peptides Synthase (NRPS) domains from DNA or amino acid sequence data [128]; NP searcher for the detection and annotation of PKS and NRPS gene clusters [129,130]; PKSIIIpred for the prediction of PKS and NRPS structures [131,132]; Structure-Based Sequence Analysis of PKS (SBSPKS) for detection and annotation of PKS and NRPS gene clusters and prediction of substrate specificity, identification of key PKS/NRPS amino acid residues, and 3D modelling of PKS modules [133,134].

Genome-guided strategies have been applied to the discovery of secondary metabolites such as PKs, NRPs and PK-NRP hybrids. These secondary metabolites are biosynthesized by large multisynthase complexes [135,136]. The genome sequence of the marine actinomycete, *Salinispora tropica*, reported by Udwary *et al.* [137] revealed a complex secondary metabolome. Bioinformatics analysis showed that a large percentage of its genome (almost 10%) was responsible for the biosynthesis of natural products, such as genes encoding for PKS and/or NRPS. Particularly, *S. tropica* was shown to produce a potent anticancer PK-NRP hybrid, salinosporamide A. After bioinformatic analysis predicted the structure of this PK-derived natural product, a polyene macrolactam with the predicted structure was isolated. A detailed overview of the biochemistry of PK pathways is referred to in other reviews [128,138,139]. In a publication by Zhang *et al.*, several secondary metabolite gene clusters were identified from the sequenced genome of *Streptomyces* sp. W007 [140]. One of these gene clusters indicated that *Streptomyces* sp. W007 may produce aromatic PK of angucyclinone antibiotics. In this way, 3-hydroxy-1-keto-3-methyl-8-methoxy-1,2,3,4-tetrahydro-benz[α]anthracene and kiamycin were isolated from fermentation extracts of *Streptomyces* sp. W007. Both secondary metabolites showed potent cytotoxicity against human cancer cell lines [140]. 

The cryptic or silent pathways, in which the putative natural product are overlooked under standard culture methods and detection conditions, are attracting the attention of the researchers as they present new opportunities for the discovery of novel bioactive compounds [141]. Genome sequencing has allowed the identification of cryptic genes in *Streptomycetes* and other microorganisms [141]. By using different strategies, it has been possible to determine the structures and bioactivities of the encoded molecules. This includes the selection for mutations in genes that enhance transcription and translation, overexpression or disruption of regulatory genes, modification of the culture medium, and overexpression of an entire biosynthetic gene cluster, gene cassettes or single cryptic genes in heterologous hosts [142]. Some examples are the biosynthesis of antibiotics, such as polyeneoic acid amide, 4-*Z*-annimycin, 4-*E*-annimycin by *S. calvus* [142], and enediynes by *Streptomyces coelicolor* and *Streptomyces avermitilis* [141].

### 3.3. Gene-Guided Bioprospecting

Gene-guided screening has been developed towards target genes associated with the biosynthetic pathways of bioactive compounds, such as those associated with the production of PKS [117,118], NRPS [117,118], bacteriocins [143] and dTDP-glucose-4,6-dehydratase [144]. For example, Wu *et al.* [144] reported the isolation, structure, and biological activities of two new 6-deoxyglycosidic elaiophylin antibiotics. PCR-based genetic screening targeting the dTDP-glucose-4,6-dehydratase gene revealed that a marine sediment derived strain, *Streptomyces* sp. 7-145, had the potential to produce glycosidic antibiotics. Guided by the PCR results, chemical investigation of one of the strains, *Streptomyces* sp. 7-145, led to the isolation and characterization of 11′,12′-dehydroelaiophylin and 11,11′-*O*-dimethyl-14′-deethyl-14′-methylelaiophylin. The former showed antibacterial activities against a number of drug-resistant pathogens, including methicillin-resistant *S. aureus* (MRSA) and vancomycin-resistant enterococci (VRE) strains.

Ansamycins are a family of macrolactams that are synthesized by type I PKS using 3-amino-5-hydroxybenzoic acid (AHBA) as the starter unit. Most members of the family have strong antimicrobial, antifungal, anticancer and/or antiviral activities. Wang and coworkers [145], through PCR screening of AHBA synthase gene, identified 26 of these genes. A similar strategy was performed by Kalan *et al.* to identify polyether epoxidase genes, a critical tailoring enzyme involved in the biosynthesis of polyether ionophores. A total of 44 putative polyether epoxidase gene-positive strains were obtained by the PCR-based screening of 1068 actinomycetes isolated from eight different habitats [146]. Hornung *et al.* [147] used PCR screening to identify 103 novel putative halogenase genes involved in the synthesis of halometabolites from 550 randomly selected actinomycetes strains. 

The screening of bacterial genome databases for genes encoding enzymes with potentially novel biochemical characteristics offers an increasingly attractive option in enzyme discovery. The most common approaches that are employed to identify enzymes in a genome are based on sequence similarity with homologs whose function is known. This is routinely performed by using either sequence–sequence comparison methods such as FASTA [148], BLAST [149], MetaGene [150], GeneMark [151] or by using profile-sequence comparison methods like PSI-BLAST [152]. This approach has been used to identified epoxide hydrolases [153], ω-transaminases [154], and nitrilases [155] among others.

The combined strategy of gene and bioactivity based screens creates a more powerful tool which allows us to obtain valuable strains with the potential to synthesize new bioactive compounds. As previously discussed, marine sponge associated bacteria have been shown to produce a cocktail of secondary metabolites [6,156]. With this in mind, a combined gene and bioactivity-based approach was used by Zhang *et al.* [157] in the identification of 15 NRPS genes from 109 bacteria obtained from four separate marine sponges. Most of the NRPS fragments identified in the study were highly diverse and potentially novel, as they displayed less than 70% sequence identity to their closest known neighbor.

### 3.4. Metagenomics

**Figure 3:**
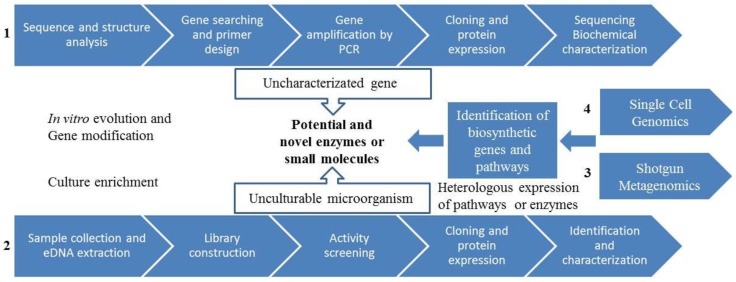
Metagenomic approaches for the discovery of novel biocatalysts or small molecules. (**1**) Sequence-based screening; (**2**) Functional-based screening; (**3**) Shotgun metagenomics and (**4**) Single cell genomics.

Metagenomics is the study of DNA from a mixed population of organisms and initially involves the cloning of either total or enriched DNA directly from the environment (eDNA) into a host that can be easily cultivated [158]. Currently, function driven analysis and sequence-driven analysis are the two main approaches used for eDNA library screening (Figure 3) [77,159]. More recently, advances in NGS technologies have allowed isolated eDNA to be sequenced and analyzed directly from environmental samples (via direct eDNA sequencing or shotgun metagenomics) (Figure 3) [160,161]. This is an effective strategy to access bioactive compounds encoded by the genomes of previously uncultured microbes through introduction of eDNA into a suitable host, bypassing the laborious step of library construction. This strategy has been used in human genome project and has also aided in the identification of novel biomass-degrading enzymes from cow rumen and compost [161].

#### 3.4.1. Metagenomics: Functional Screening

Functional screening has allowed for the identification of a large number of bioactive compounds and biosynthetic pathways including industrially important enzymes such as cellulases [21], proteases [162,163], lipases [164,165], esterases [166,167,168], glycoside hydrolases [19]. For example, the isolation of clone-specific metabolites produced by eDNA clones identified from bacterial top agar overlay assays has led to the characterization of a wide variety of bioactive compounds. Among these compounds can be found long-chain *N*-acyl amino acid antibiotics, a novel isonitrile functionalized indole antibiotic, antibacterial active pigments such as violacein, indigo and turbomycin [169], terragines [170], fatty acid derivatives [171], and aromatic poliketydes [172], all of which have been recovered from soil libraries. The cyclic peptides nocarmide and patellamide D have also been isolated in this manner from soil and marine sponge libraries, respectively [173,174]. Functional screening was used by Selvin *et al.* [164] for the discovery of novel lipases. In this manner, a marine metagenomic library was built from the DNA extracted of the sponge *Haliclona simulans.* This library of 48,000 clones was screened by plates supplemented with tributyrin. Using this approach, a novel lipase was identified, heterologously expressed and characterized. In another study, Robertson *et al.* [175] collected 651 environmental samples from terrestrial and aquatic microenvironments. Fragments of these samples 1–10 kb in size were cloned into genomic eDNA libraries. Authors primarily isolated inserted DNA fragments containing target genes using a culture dependent assay with adiponitrile or (*R*,*S*)-4-chloro-3-hydroxyglutaronitrile as the sole nitrogen source. Growth resulted from a clone’s ability to hydrolyze the nitrile substrate, generating ammonia, which enabled the clone’s growth. Positive clones were isolated and sequenced for subcloning. Finally, 137 unique nitrilase genes were expressed and characterized. A similar approach was used by Bayer *et al.* [176] to select nitrilases from metagenomic libraries (one from oil contaminated soil, two from forest soils and one from gravel from an experimentally constructed wetland for wastewater treatment from a refinery) resulting in the isolation of *Nit1*, an aliphatic nitrilase which catalyzes dinitriles.

#### 3.4.2. Metagenomics: Sequenced-Based Screening

Sequenced-based screening using homologous PCR or clone hybridization allows the identification of essential genetic components for cluster assembly. Sequenced-based screening has been used together with NGS technologies in the identification of PKS and NRPS clusters from a number of cultured strains [177]. Further to this, sequence-based analysis of marine metagenomic libraries has to date revealed the presence of a number of enzymes including peptidases [178], alkane hydroxylases [179], laccase [15], and a fumarase [17]. In the case of the peptidases, the Sargasso Sea Whole Genome Sequence (WGS) dataset, involving a total of 1.045 billion base pairs of metagenomic DNA sequence, with over 1.2 million protein encoding ORFs [180], was analyzed by Cotrell and coworkers [178] by using BLAST, in the identification of potential hydrolases. Following cloning and expression in the heterologous *E. coli* host, an abundance of peptidase was detected. Bayer *et al.* [181] designed degenerate PCR primers to identify FADH_2_-dependent halogenase genes from several Caribbean and Mediterranean sponges. By sequence-based screening, three novel phylogenetically and functionally distinct halogenase gene clusters were discovered. Another example of this approach appears in a recent report on the isolation of a novel laccase from a marine microbial metagenome of the South China Sea [15]. Here, researchers used PCR primers based on the conserved copper binding sites to identify putative laccases in metagenomic DNA. Subsequently, the gene encoding the putative laccase was heterologously expressed in *E. coli*, yielding a recombinant protein that exhibited alkalescence-dependent and chloride- tolerant laccase activity.

Another interesting approach involves the *in silico* screening of NCBI sequence databases, for example, using bioinformatics, and the subsequent identification of putative genes of interest. Genes identified using this approach are usually uncharacterized, annotated as being either putative or hypothetical, or are from either environmental metagenomes or unknown organism sources. These genes are then computationally optimized for expression in suitable hosts, and the DNA is chemically synthesized, cloned into expression vectors and functionally screened in heterologous systems, such as *E. coli* or *Saccharomyces cerevisiae*. Such an approach has been successfully employed in the identification of methyl halidetransferases (MHT), with potential applications in the production of methyl halides from different biomass sources, thereby transforming renewable carbon sources into products such chemicals and liquid fuels [182]. Vergne-Vaxelaire *et al.* [155] collected sequences coding for experimentally characterized nitrilases and used these for similarity sequence analyses using UniprotKB and Genoscope databases. Candidate nitrilases were then selected according to their genome availability in the Genoscope strain collection and from a wastewater treatment plant metagenomic library. From the 290 selected candidates, 163 genes were successfully cloned and overexpressed in *Escherichia coli* BL21. While only 9.6% of these enzymes were previously annotated as predicted nitrilases, 17% were misannotated as amidohydrolases and the majority of the remainder of enzymes were only annotated as putative carbon-nitrogen hydrolases. These putative enzymes were screened against 25 nitriles chosen to represent a wide structural diversity (such as β-hydroxylated, α-hydroxylated, β-aminated, arylaceto-, saturated and unsaturated nitriles). Out of the 125 studied nitrilases, 31 were purified and characterized for substrate specificity. With a selection of these nitrilases, the authors performed the synthesis of three building blocks (such as 4-methoxy-4-oxo-3-phenylbutanoic, 3-oxocyclopentanecarboxylic acid and 2-((cyanomethyl)aminoacetic acid)) which are difficult to synthesize using conventional organic methods. The availability of these enzymes with nitrilase activity opens the way for the improvement of their catalytic properties by genetic engineering. This collection of nitrilases could be very useful in the production of nitrile derivatives as building blocks.

#### 3.4.3. Novel Metagenomics Approaches

In contrast with the conventional metagenomic approach, single cell genomics (Figure 3) begins with the isolation of the microbial cell fraction from an environmental sample and the separation of an individual prokaryotic cell through microfluidics, cytometry, or micromanipulation. Single cell genomics is dependent on multiple displacement amplification (MDA), which allows the generation of genomic DNA suitable for shotgun sequencing from unique microbial cells. Thereby, the entire biochemical potential of a single uncultured microbe can be evaluated from within a complex microbial community [183]. Using this approach, microbial cells are firstly singularized by FACS, sorted, and individual microbial cells are then subjected to Whole Genomic Amplification (WGA). The amplified genome can then be sequenced, and the catalytic and metabolic potential of the genome analyzed. This approach has recently been employed by the Hentschel group, who used single cell genomics to characterize Poribacteria that form part of the microbial consortia of the Mediterranean sponge *Aplysina aerophoba* [184]. Using this approach, nearly 1.6 Mb of DNA sequence was obtained from the poriobacterial genome that, following the annotation, allowed the identification of several enzymes, including several sulfatases and peptidases.

Recently, there has been a huge increase in the development of novel screening methods based on metagenomic tools. For example, Substrate-Induced Gene-Expression screening (SIGEX), metabolite-regulated screening (METREX) and a technique based on subtractive hybridization magnetic bead capture. METREX involves the introduction of metagenomic DNA into a suitable host cell containing a biosensor plasmid to detect compounds of interest, for example, compounds that induce bacterial quorum sensing. Thereby, a sensor such as green fluorescent protein (GFP) is expressed by the cell either when gene (or genes) is introduced into the cell or when an exogenous quorum-sensing inducer is applied leading to the synthesis of an inducer. This can be identified using FACS or by fluorescence microscopy [185]. SIGEX has been developed to isolate novel catabolic genes from metagenomes which are particularly difficult to obtain using traditional gene cloning methods [186]. In SIGEX, digested metagenome fragments are ligated into an operon-trap vector containing a reporter protein such as GFP, and a metagenomic library is constructed in a liquid medium by transforming a suitable cloning host. This library is screened by a SIGEX assay, and positive clones are selected by detecting the activity of a co-expressed marker, in this case, the GFP fluorescence. [187]. Another interesting approach that has been described is based on substrate hybridization capture involving the use of magnetic beads [188]. This involves the amplification of the internal portion of the gene of interest using degenerate primers, and the subsequent immobilization of the partial gene amplicons on streptavidin-covered magnetic beads. These beads are then used as hybridization probes to target full-length genes from metagenomic DNA. This method has been used to clone novel bacterial multicopper oxidases from soil but would clearly also have use in marine metagenomic samples [189]. Another example has been described by Margassery *et al.* [190], where HTS was used to identify calcineurin inhibitors in large libraries of chemical compounds, microbial extracts and metagenomic libraries. Calcineurin is a eukaryotic calmodulin-dependent serine/threonine phosphatase type 3CA involved in several neurodegenerative diseases. There is a large amount of interest in identifying novel calcineurin inhibitors due to side-effects that occur with the long-term use of those currently available. This HTS-directed approach is based on the detection of activity of a *CDRE::lacZ* gene fusion in pMRK212 plasmid introduced into *S. cerevisiae*. A cytoplasmic calcium concentration is triggered by alkaline pH, which activates calmodulin, calcineurin and Crz1p (the main target of calcineurin in yeast). Dephosphorylation by calcineurin causes Crz1p translocation to the nucleus, where it activates transcription of target genes by binding to a promoter sequence known as the CDRE element. Expression of the *CDRE::lacZ* gene fusion can be detected using a modified enzymatic assay for the β-galactosidase. The validation of this method was carried out by the HTS of extracts of marine sponge-associated bacteria. In this way, Margassery and coworkers identified three candidate extracts with potential calcineurin inhibitor [190]. To demonstrate that calcineurin prevents the translocation of Crz1p to the nucleus, the construct *GFP::CRZ1* gene fusion was used. By fluorescence microscopy, GFP fluorescence was observed throughout the cell. This, therefore, supported the theory that calcineurin prevents the Crz1p translocation to the nucleus. Thereby, this novel HTS approach proved successful in the identification of potential calcineurin inhibitors.

### 3.5. Combinatorial Biosynthesis

Combinatorial biosynthesis is a technology based on the genetic manipulation of biosynthetic clusters encoding bioactive compounds. Genetic manipulation techniques used include amino acid substitution, deletion or inactivation, swapping or borrowing of genes within a module, gene fusions, and assembly of these components, with the aim of producing novel structures in order to obtain new or altered structures that would be difficult to synthesize using other methods [191]. This approach has been used mainly in multi-modular enzymes such as PKS, NRPS and hybrid PKS-NRPS. The rapid advances in microbial genome analysis have not only enabled the identification of these gene clusters, but have also provided the necessary tools for engineering the biosynthesis of novel compounds by combinatorial biosynthesis [139,191,192,193]. 

For example, Kim *et al.* obtained a barbamide biosynthetic gene cluster from the marine cyanobacterium *Moorea producen*, and subsequently heterologously expressed it in a genetically engineered strain of *S. venezuelae* DHS 2001 where the pikromycin PKS gene cluster was deleted. This approach resulted in the production of the marine natural products, 4-*O*-demethylbarbamide, a barbamide derivative [194]. Another example has been described by Doekel and coworkers for the production of analogues of the antibiotic daptomycin [195]. The lipopeptide backbone of daptomycin is produced by three NRPS multi-synthases encoded by *dptA*, *dptBC* and *dptD* genes, which are interconnected by peptide linkers. Authors introduced mutations such as amino acid substitutions, insertions and deletions in the inter-module linkers with no negative effects on the lipopeptide production. dptD enzyme consists in two modules—one of the modules incorporates 3-methylglutamic acid (3mGlu_12_) and the other incorporate kynurenine (kyn_13_) to the daptomycin. Daptomycin was redirected to incorporate Trp and Ile/Val, in place of Kyn_13_, as position 13 has been described as crucial for determining the antimicrobial activity of the antibiotic. In this way, hybrids dptD were constructed by fusion of 3mGlu_12_ to the terminal modules for Trp_11_ and Ile_13_ from CDA (Calcium Dependent Antibiotic) and A54145 NRPSs, respectively. Lipopeptide biosynthesis was restored in strain *S. roseosporus* UA378 with dptD deleted in these hybrid subunits, resulting in similar efficiency to those recombinants reconstructed to produce daptomycin. These hybrid recombinant cells produced daptomycin analogues with Trp_13_ or Ile_13_ at high efficiencies. Moreover, a recombinant dptD strain with a hybrid Kyn_13_ module synthesized a novel daptomycin analogue containing Asn_13_.

This approach has also been used for the production of antimycins (ANTs). ANTs differ in their alkylation at C7 and acylation at C8, however, the effects of these functionalities on biological activities and associated modes of action remain unclear [196]. The *antB* gene was deleted in the ANT-producing strain *Streptomyces* sp. NRRL 2288, aiming at the construction of an engineered biosynthetic apparatus for diversity-oriented production of dilactone scaffolds *in vivo* [197]. The resulting mutant strain AL2110 failed to produce mature ANTs, but accumulated a series of C8-deacylated ANTs that vary in the alkylation at C7. Three carboxylates, chloropentanoate, cyclohexanepropanoate, and 10-undecynoate, were then fed to AL2110 strain, to examine whether naturally unavailable units could be incorporated into ANTs to increase the diversity at C7. As a result, all feedings produced new C8-deacylated ANTs [197]. Another example is the production of the fluorinated analog fluorosalinosporamide by engineering the bacterium *Salinispora tropica*. *S. tropica* has been modified by replacing the chlorinase gene *salL* by the fluorinase gene *flA* from *Streptomyces catteleya* [198]. Thereby, *salL^−^flA*^+^ mutant strain in the presence of inorganic fluoride induced the production of fluorosalinosporamide.

Despite the many successes of combinatorial biosynthesis, engineered biosynthetic clusters often show lower catalytic efficiency (and hence lower productivity) than the original cluster [191].

### 3.6. Synthetic Biology

Synthetic biology is a promising strategy to improve the production of known marine compounds or activate silent gene clusters by genetic manipulation of the biosynthetic machinery (natural or artificial) involved in the assembly of bioactive compounds [199]. This approach is based on the development of genome manipulation techniques such as hierarchical Conjugative Assembly Genome Engineering (CAGE) [200] and Multiplex Automated Genome Engineering (MAGE) [201], as well as functional characterization of abundant genetic materials (e.g., controllable regulatory elements, synthetic RNA/protein scaffolds) [202]. In addition to the efficient genome handling and transfer technologies, compatibilities between microbial hosts and all the necessary machinery to obtain the targeted product are very important for the choice of the most suitable host. This includes the proper expression of the genes responsible for the production of the target compound, compatibility with the enzyme activity and the availability of precursors. Nowadays, synthetic biology is used for the development of microbial cell factories for bioactive compound production [203] or to synthesize gene clusters enabling *in situ* therapeutic delivery [202]. In recent years, many successful examples of bioactive compounds production with therapeutic interest through synthetic biology have been reported. Antibiotics such as aminoglycosides derivatives, which include neomycin, kanamycin and gentamicin [204], as well as other natural products like PKS [205,206,207] and NRPS [207] have been produced. In addition, the enzymes responsible for the production of these chemical compounds can be isolated and used as biocatalysts to synthesize bioactive compounds, their intermediates, and derivate compounds.

An interesting approach is the retrobiosynthetic method, which is based on the backward search for biosynthetic routes to a target a compound such as a bioactive compound or an enzyme. Through the implementation of a defined set of biochemical transformation rules (namely retrosynthesis algorithms, such as pathway length, favorable thermodynamic, estimate gene compatibility, estimate enzyme performance, and compound toxicity) efficient heterologous biosynthetic metabolic pathways are designed and inserted in host organisms [208]. A retrosynthetic map containing the entire metabolic network, including annotated and putative enzymatic reactions catalyzed by identified enzymes, and alternative metabolic pathways to reach the target compound can then be designed. All the necessary information to build a retrosynthetic map can be found in various databases, such as Kyoto Encyclopedia of Genes and Genomes (KEGG) which gives information about enzyme sequences and annotated reactions, and BRaunschweig ENzyme Database (BRENDA) which provides experimental enzymatic kinetic constants. All this information provides a full biosynthetic automated network for the design and production of bioactive compounds and enzymes, considering the insertion cost of each metabolic pathway based on different criteria such as gene insertion cost, expression levels and catalytic efficiency [208]. This novel approach is attracting the attention of biocatalysis [209] and is allowing the identification of previously unknown biosynthetic routes, such as some antimicrobial peptide biosynthetic routes [207].

Another novel approach that allows the transfer of a complete biosynthetic pathway into a different bacterium has been reported recently by Loeschcke and coworkers [210]. The TRansfer and EXpression system (TREX) involves the transfer an entire biosynthetic pathway to a suitable host. TREX employs conjugation for DNA transfer, randomized transposition for its chromosomal insertion, and T7 RNA polymerase for unhindered bidirectional gene expression. The TREX system consists of two cassettes, designated left and right (L-TREX and R-TREX), which contain all functional elements needed for establishing a novel biosynthetic pathway in a bacterial host. In addition, L-TREX and R-TREX cassette contain selection markers, namely tetracycline resistance gene and gentamicin resistance gene, respectively. The target gene cluster (which can be located on BAC, plasmid or cosmid) is labeled by the two TREX cassettes. The transfer of metabolic pathways can be achieved by restriction endonuclease-based cloning or more advanced techniques such as restriction enzyme-independent cloning, and recombineering techniques based on λ phage or yeast recombinases that enable a better handling of large DNA fragments [210]. In the second step, the TREX-labeled gene cluster is transferred to a bacterial host. The origin of transfer, located into L-TREX, enables the conjugational transfer of large DNA fragments. This fact allows the efficient release of recombinant DNA molecules. In the next step, the gene cluster is inserted into the chromosomal DNA of the expression host by transposition. Finally, two T7 RNA polymerase-dependent promoters, located within the L- and R-TREX cassettes in opposite directions, allow the bidirectional expression of cluster genes regardless of their orientation. The authors constructed a TREX module, namely <L-TREX-R> to simplify the TREX labeling. This module—provided on plasmid pIC20H-RL, GenBank Accession Number: JX668229—includes both TREX cassettes where the two T7 promoters point outwards. This system has been applied to the transfer and expression in bacteria of two biosynthetic pathways responsible for the production of two secondary metabolites, prodigiosin and zeaxanthin [210]. In this way, TREX can help to identify and synthesize novel bioactive compounds.

Therefore, synthetic biology permits the user manipulate the cell biosynthetic machinery to produce unnatural metabolites, to improve the production of known compounds and to activate silent gene clusters of biosynthetic pathways. Synthetic biology is an emerging discipline that, upon further development, will play an important role in the production of marine bioactive compounds [211].

### 3.7. Heterologous Production of Bioactive Compounds

In order to achieve the heterologous production of novel bioactive compounds, the development of marine-derived hosts such as cyanobacteria, actinomycete, and symbiotic fungi is key. Moreover, heterologous expression of genes or entire biosynthetic gene clusters involved in the synthesis of bioactive compounds is an important strategy in the identification of the function of these genes or genes clusters [212]. Techniques such as natural product screening, compound chemical characterization, host isolation, gene cluster identification, sequencing analysis or synthesis, and metabolic and process engineering are necessary in the optimization of heterologous production [213]. 

Through heterologous production, compounds such as PKs in *E. coli* (6-methylsalicylic acid and 6-deoxyerythronolide B) [213,214], granaticin, epothilones A, 6-deoxyerthronolide B, medermycin, 6-methylsalicylic acid, novobiocin and several type II PK products in *Streptomyces coelicolor* [215,216,217,218,219,220,221,222], and 6-methylsalicylic acid in *S. lividans* [223] have been obtained. Also, heterologous production of NRPs and hybrid NRP-PKs compounds has been performed in *Streptomyces* spp (daptomycin and capreomycin) [224], in *E. coli* (echinomycin, yersiniabactin, tyrocidine intermediates) [225,226,227,228], *Bacillus subtilis* [213], and *Pseudomonas putida* [229]. As well as this, the heterologous production of isoprenoids (such as artemisin and taxol) has been described in *E. coli* and *S. cerivisiae*, respectively [193,213]. 

#### 3.8. “Omic” Integrated Approaches to Characterize Bioactive Compounds Biosynthetic Gene Clusters and Pathways

“Omics” approaches utilize genomic, proteomic, metabolomic, and transcriptomic tools to bypass cultivation limitations by studying the collective material of organisms from environmental samples, thus enabling powerful new approaches to gene, genome, protein and metabolic pathway discovery [161]. Access to NGS and the development of bioinformatics tools is essential for the resolution of the tremendous databases generated from these technology applications. Below, we will discuss in detail case studies where integrated “omic” approaches were used successfully in the discovery and characterization of biosynthetic gene clusters and pathways. 

##### 3.8.1. Integrated Approach to Investigate the ET-743 Biosynthetic Pathway

**Figure 4:**
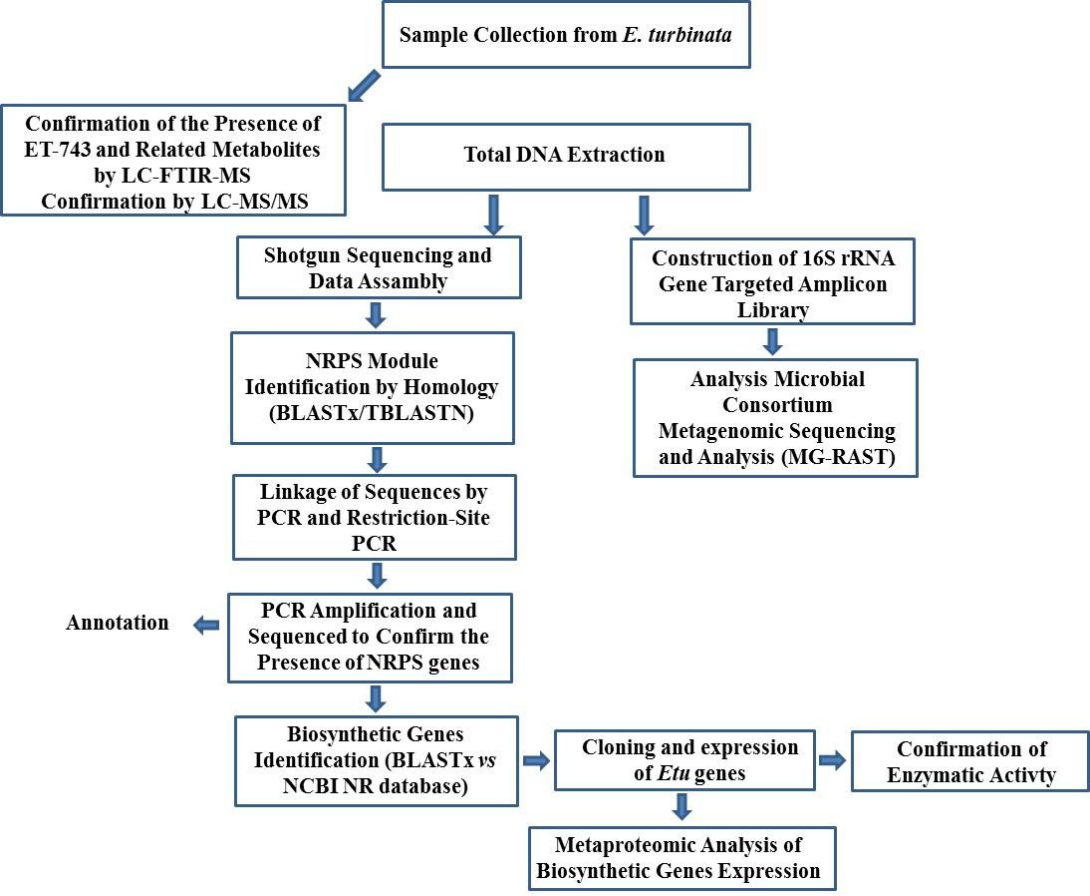
An integrated approach to investigate the ET-743 biosynthetic pathway.

An integrated “omic” approach was used by Rath *et al.* [230] to identify and characterize the ET-743 or trabectedin (Yondelis^®^) biosynthetic pathway from invertebrate-derived microbial consortia. A summary of this approach taken by Sherman *et al.* can be seen in Figure 4. ET-743 is a tetrahydroisoquinoline with strong anticancer activity isolated from the tunicate *Ecteinascidia turbinata.* This compound has been clinically approved in Europe against sarcoma and ovarian neoplasms. In this study, ET-743 was produced semi-synthetically for clinical application from fermentation-derived cyanosafracin B. Raw reads from the shotgun sequencing were assembled and subsequently filtered using BLASTX and TBLASTN to measure the relatedness of translated protein sequences to safracin and saframycin NRPS. Twenty-five presumed ET-743 biosynthetic genes were identified and annotated with proposed functions that account for all of the core NRPS genes of ET-743 using the BLASTX tool against the NCBI NR database. These results suggested that individual genes appear to be of bacterial origin (for example, lacking introns and polycistronic) and hence the gene cluster is not derived from the tunicate genome. The presence of the predicted enzymes from the sequence analysis involved in the ET-743 biosynthetic pathway was used to mine the metaproteome by mass spectrometry. ET-743 biosynthetic pathway enzymes predicted via gene cluster analysis were then searched for in the metaproteome using mass spectrometry. 

16S rRNA gene amplicon library and a random shotgun fragment library for 454 pyrosequencing were prepared using this DNA. Additionally, MG-RAST was used to perform the taxonomic classification of the raw reads and of the total assembly. Results showed that ~40% of the classified sequences were of eukaryotic origin (mainly *Ciona*), and 60% were of proteobacterial origin, within which the two major populations were γ-proteobacterial and α-proteobacterial. Sequence analysis based on codon usage of two large unlinked contigs suggested that *Candidatus Endoecteinascidia frumentensis* produces the ET-743 metabolite. Finally, the expression of three key biosynthetic proteins, along with the functional analysis of these enzymes, confirmed their assigned catalytic activity in the biosynthetic pathway, enabling the direct correlation between bioactive compound, the *Etu* gene cluster, and predicted biosynthetic enzymes. Therefore, these three biosynthetic pathway enzymes were identified, and comparisons with standards suggested that ET-743 biosynthetic genes expressed in the tunicate-microbial community. These *in vitro* findings will drive future efforts to engineer the production (via heterologous expression and synthetic biology) of the ET-743 drug and its related analogues. Moreover, this strategy provided a general approach to characterize bioactive compound biosynthetic systems from complex marine consortia. 

##### 3.8.2. An Integrated Approach in the Discovery a Novel Lantibiotic from *B. subtilis* Strain Isolated from a Marine Sponge

**Figure 5:**
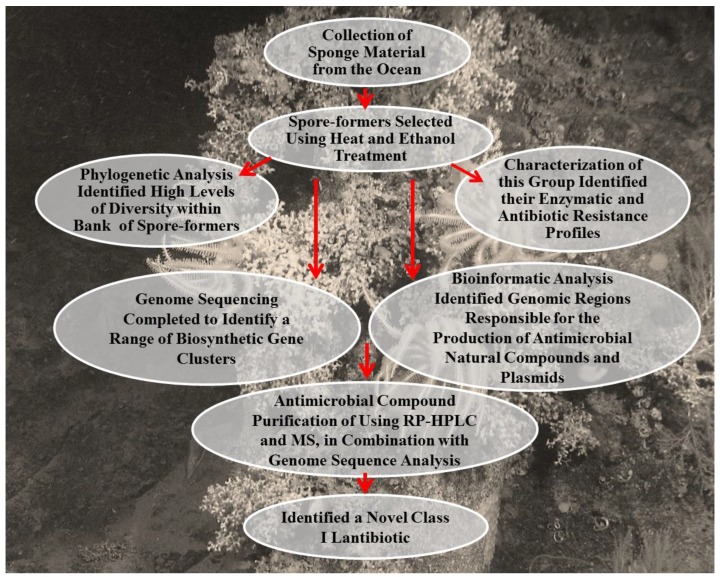
Bioprospecting of novel natural compounds. A combined approach of traditional assays and modern genomic technologies were utilized by Phelan *et al.* to unlock the biotechnological potential of the marine sponge associated endospore-formers.

An interesting approach has been recently used in the discovery of subtilomycin, the novel lantibiotic from *B. subtilis* MMA7 isolated from the marine sponge *Haliclona simulans* (Figure 5). This approach combined traditional analysis with modern genomic technologies [231]. Antimicrobial assays were performed with a colony overlay assay, which demonstrated activity against several bacterial strains such as marine associated Gram-negative bacteria and pathogenic strains. Conventional PCR was used to test for the presence of genes encoding bacteriocins such as subtilin, subtilosin and sublancin in *B. subtilis* MMA7. Only the presence of subtilosin was detected by PCR. However, Phelan and coworkers did not observe any differences in antimicrobial activity between the wild type and MMA7 strain after inserting a mutation in the subtilosin biosynthetic gene cluster. This result suggested that other antimicrobial compounds were being produced by this strain. In order to purify this unknown compound, the active microbial crude extract was purified by Reverse Phase High Performance Liquid Chromatography (RP-HPLC). The active fractions collected from RP-HPLC were subjected to MALDI-TOF MS, which revealed a unique peak corresponding to a mass of 3235 Da. After a search of different antimicrobial databases, no matches were found with this molecular mass. When tested, the compound retained antimicrobial activity against indicator strains used in the initial antimicrobial assays. Primary amino acid sequence was obtained using trypsin digestion and alkylation of this peptide, followed by MALDI MS and tandem mass spectrometry analysis (MS^2^). The obtained amino acid sequence was used in BLASTP and BLASTN searches against the genome of *B. subtilis* MMA7 to look for genes involved in the synthesis of this peptide. In this manner, the gene encoding the novel class I lantibiotic subtilomycin was identified by coupling functional based screening assays with an integrated “omic” approach. 

##### 3.8.3. Integrated Approaches in the Investigation of the Biosynthetic Pathways and the Characterization of Glycosylated Bioactive Compounds

Glycosylated natural products (GNPs) are a structurally different class of molecules with important agrochemical and pharmaceutical applications [232]. Kersten and coworkers characterized the *O*- and *N*-glycosyl groups in their sugar monomers by MS*^n^* analysis and connected the groups to their corresponding genes in secondary metabolic pathways through an MS-glycogenetic code [232]. Firstly, the extract of sequenced bacteria was analyzed by LC-MS*^n^* to detect the presence of GNPs. The candidate GNPs were then identified according to the peaks of extracted ion chromatograms (sugar-specific B/C-ion masses or Y/Z-ion neutral losses) in the chromatogram data. These putative GNPs were confirmed by characterization of the MS*^n^* spectra. Subsequently, the putative GNP molecules were connected with the corresponding glycosylation genes in the microbial genome by genome mining, based on the observed sugar fragments. On the other hand, the glycogenomic approach was used to test the presence of new glycosylated compounds in *Salinispora arenicola* CNB-527 and *Streptomyces* sp. SPB74. The genome sequences of these bacteria were analyzed by antiSMASH. All the secondary metabolic gene clusters were predicted and analyzed for the presence of specific and widespread glycosylation genes, in addition to a functional prediction of glycosylation genes by BLAST. In this manner, the presence of glycosylation genes was tested in each observed MS*^n^* candidate sugars, and the positive matches were analyzed by BLAST. The structure of these positive matches was elucidated from MS*^n^* and genetic data. Finally, a further characterization of the purified compounds was carried out by NMR. This approach led to the rapid characterization of the anticancer agent cinerubin B and its gene cluster from *Streptomyces* sp. SPB74, and the discovery of the antibiotic arenimycin B, and its biosynthetic gene cluster from *S. arenicola* CNB-527. 

Therefore, MS*^n^* can be used in the analysis of microbial metabolic extract to identify biosynthetic building blocks. Used together with genome mining, MS*^n^* can be aimed at a number of expressed biosynthetic pathways in one assay. In addition, combined with automated platforms like liquid chromatography-tandem mass spectrometry (LC-MS), MS*^n^* analysis has the potential to be automated.

##### 3.8.4. Integrated Approaches using Orthogonal Active Site Identification System (OASIS) and Proteomic Interrogation of Secondary Metabolism (PrISM)

The Orthogonal Active Site Identification System (OASIS) and Proteomic Interrogation of Secondary Metabolism (PrISM) have had a significant impact on the application of proteomic to natural products research [161]. 

PrISM allows for the screening of the expressed enzymes related to natural product biosynthesis [233]. In a PrISM, microbes are cultured under various conditions, and their proteomes are analyzed by MS. Expressed proteins for secondary metabolite biosynthesis are detected, which enables the biosynthetic gene cluster and the associated secondary metabolite to be discovered simultaneously. The PrISM approach was used to screen expressed NRPSs and PKSs from *Streptomyces* spp. and to identify an orphan NRPS gene cluster from *Streptomyces* sp. NRRL F-6652. Through bioinformatics analysis of the gene cluster and metabolomics analysis using MS, a new class of peptide aldehyde natural products called flavopeptins was discovered and identified as the products of the orphan gene cluster [234]. These flavopeptins are synthesized through an NRPS and contain a terminal NAD(P)H dependent reductase domain probably used for the reductive release of the peptide with a C-terminal aldehyde. Similar to other peptide aldehydes, flavopeptins showed inhibitory activities against cysteine proteases and anti-proliferative activity against multiple myeloma and lymphoma cell lines [234]. Another example of this strategy is the discovery of koramine (an NP associated with *Bacillus* species) through the PrISM method [235] described by Evans *et al.* [235]. Evans and coworkers cloned the complete gene cluster and elucidated the biosynthesis of the novel compound, which is produced by a non-sequenced *Bacillus* sp. strain isolated from soil. The original discovery of the compound began with a proteome strategy, where microbial proteomes were scanned for expressed gene clusters. 23 unsequenced environmental isolates were cultured under nine different sets of conditions and analyzed by SDS-PAGE. Of the 23 strains, 20 showed some evidence of proteins over 150 kDa in size. These tend to be large NRPS or PKS enzymes, which often are >1300 amino acids in length. A MS analysis of direct peptide sequencing by MS^2^ gave the amino acid subsequences that guided the design of PCR primers for amplification of DNA corresponding to the region between the identified peptides and the conserved core regions of NRPS adenylation domains. Eventual DNA sequencing of this >30 kb gene cluster and prediction of its functional elements led to the prediction and the detection of a new peptide natural product named koranimine. The structure of koranimine was determined using multistage MS^2^, stable isotope incorporation, NMR spectroscopy, and *in vitro* enzyme reconstitution [235]. 

OASIS uses chemical probes that bind the active sites of NRPS and PKS enzymes allowing the enrichment of complex proteomic samples before being applied to MS^2^ [236]. These enzymes utilize a small carrier protein (CP) to covalently bond activated monomers and intermediates directly to the enzymes throughout the biosynthetic process domain. The site of this covalent attachment is at the terminal thiol of the 4′-phosphopantetheine (PPant) prosthetic group. The PPant posttranslational modification has only been observed within PKS, NRPS and related enzymes, and their activities can be detected through labeling approaches (e.g., enzyme inhibitors labeled with fluorescence) or observation of a unique fragmentation during MS^2^. This approach has been used to obtain a profile of PKS and NRPS enzymes present in *Bacillus subtilis* [237].

## 4. Dereplication Strategies

**Figure 6:**
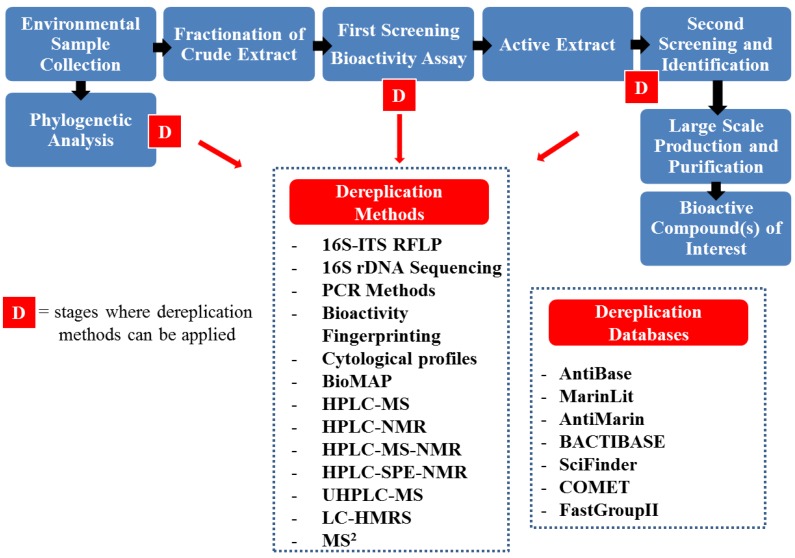
Example of general dereplication workflow. For example, the obtained MS spectra can be compared against natural products databases.

It is of the utmost importance that studies in the discovery of novel marine bioactive compounds, such as antimicrobial compounds, should be performed in parallel with a defined dereplication strategy to screen for previously known bioactive compounds. Dereplication itself is the process of screening samples for the presence of active compounds which have already been studied, thereby eliminating them from consideration. The identification of known molecules early in the marine bioactive compound discovery process minimizes time, effort and cost (Figure 6) [121,238]. Dereplication processes are generally achieved through morphological comparison of obtained colonies on different solid media, and/or using molecular methods such as 16S-Internal Transcribed Spacer (ITS) RFLP, partial 16S rDNA sequencing, and repetitive extragenic palindromic-PCR of the BOX DNA element (BOX-PCR). While these approaches have proven to be of great utility, more modern systematics-guided methods of bioprocessing and dereplication have been developed. At present, dereplication strategies include High Performance Liquid Chromatography-Mass Spectrometry (HPLC-MS), HPLC-Nuclear Magnetic Resonance (NMR), HPLC-NMR-MS, HPLC-Solid Phase Extraction (SPE)-NMR, and Ultra High Performance Liquid Chromatography (UHPLC)-MS, or bioactivity fingerprints, such as cytological profiling or BioMAP are commonly in use [238,239]. The development of high quality libraries, composed of marine microorganisms, provide a diverse collection of structurally distinct molecules to enhance the discovery of novel bioactive compounds, thereby improving the results of dereplication strategies [90]. MS can be used to confirm the size of different natural compounds, and their subsequent molecular weights can be compared against natural product databases. Nowadays, multiple databases are available, such as AntiBase, MarinLit, AntiMarin (which contains the combination of the compound data from AntiBase and MarinLit), Beilstein Dictionary of Natural Products, FastGroupII (available on [240]), SciFinder and BACTIBASE (available on [241]), a data repository of bacteriocin natural antimicrobial peptides [238,242,243,244]. Another useful tool, COMET, has been developed by Microbial Screening Technologies. COMET is a metabolite recognition software that compiles and analyzes co-metabolite patterns in natural product mixtures [245]. These resources are used to determine the novelty and proposed structure of a specific sample, thereby helping to identify the compound and ensure dereplication of previously known compounds. Despite these alternatives, the creation of an open access database of the MS spectra of known compounds would prove invaluable for researchers.

Recently, Yang *et al.* [238] and Forner *et al.* [239] used a dereplication approach involving Liquid Chromatography–High Resolution Mass Spectrometry (LC–HRMS). Only small-scale fermentation extracts are necessary to capture novel secondary metabolite production in a rapid and robust process, while minimizing the detection of undesired media components. In this way, a chemical barcode of the fermentation extract is generated. Therefore, the preprocessing of LC–HRMS data is a critical step in this study. For example, cluster analysis applied to barcoded LC–HRMS data was shown to be a sensitive and reproducible technique for the grouping of *Streptomyces* isolates according to their chemical fingerprints [239]. In this study, a large library of 120 isolates of Actinomycetes was collected from three sites off the coast of Canada. Twenty-two *Streptomyces* isolates of these 120 were selected due to their close genetic relationship despite their different geographic location. These isolates were characterized using 16S rDNA sequencing combined with BOX-PCR, and 16S-ITS RFLP. Using these molecular techniques, groups were created and compared by LC-HRMS dereplication methodology. A second group of 120 isolates was submitted to the same molecular dereplication methodology and chemically dereplicated into 35 clades to further validate this approach, and using these techniques some groups showed putative novel chemistry, as barcodes showed metabolites with unique *m/z* ratios. Forner and coworkers demonstrated that the chemical diversity of produced metabolites was reproducible and provided an enhanced resolution for the discrimination of redundant microbial strains compared to present molecular dereplication techniques. Moreover, this method provides us with the ability to identify putative novel chemical compounds.

## 5. Conclusions

The blue technology sector is a rapidly growing area, with the market projected to exceed €3.11 billion by 2015 [246], and the global market of enzymes projected to reach $4.4 billion by 2015 [246]. The discovery of novel bioactive compounds and biocatalysts is thereby hugely important and is still presenting a major challenge to researchers. As this review has shown, there is a growing arsenal of methodologies available in the field of marine biodiscovery. In the coming years, the development of emerging “omics” strategies such direct sequencing of eDNA, single cell technologies, metaproteomic and synthetic biology (such co-selection-MAGE strategy [247]) will improve the discovery and production of these compounds. With the development of model microbial systems for heterologous expression, NGS technologies (which are becoming increasingly accessible) and bioinformatic tools, the rapid exploitation of biosynthetic gene clusters will continue to be facilitated. These emerging strategies in many cases allow us to study the biosynthetic pathway systems of organisms previously inaccessible to us by traditional methods. The combination of conventional and innovative approaches, along with the use of novel dereplication strategies will continue to provide us with essential tools in the discovery of novel marine bioactive compounds with potential applications in both medicine and industry.
